# Risk Factors of Acute Rejection: Impact on Graft Outcomes in a Cohort of Kidney Transplant Recipients

**DOI:** 10.3390/jcm14103373

**Published:** 2025-05-12

**Authors:** Valeria Corradetti, Elisa Gessaroli, Federico Bari, Claudia Bini, Valeria Grandinetti, Angelodaniele Napoletano, Vania Cuna, Valeria Pizzuti, Marcello Demetri, Matteo Ravaioli, Michele Provenzano, Gaetano La Manna, Giorgia Comai

**Affiliations:** 1 Nephrology, Dialysis and Kidney Transplant Unit, IRCCS Azienda Ospedaliero-Universitaria di Bologna, 40139 Bologna, Italy; valeria.corradetti@aosp.bo.it (V.C.); claudia.bini@aosp.bo.it (C.B.); valeria.grandinetti@aosp.bo.it (V.G.); angelodaniele.napoletano@aosp.bo.it (A.N.); vania.cuna@aosp.bo.it (V.C.); valeria.pizzuti@aosp.bo.it (V.P.); gaetano.lamanna@unibo.it (G.L.M.); giorgia.comai@aosp.bo.it (G.C.); 2 Department of Medical and Surgical Sciences (DIMEC), Alma Mater Studiorum-University of Bologna, 40126 Bologna, Italy; elisa.gessaroli5@unibo.it (E.G.); federico.bari2@studio.unibo.it (F.B.); marcello.demetri2@unibo.it (M.D.); matteo.ravaioli6@unibo.it (M.R.); 3 Hepato-Biliary Surgery and Transplant Unit, IRCCS Azienda Ospedaliero-Universitaria di Bologna, 40139 Bologna, Italy; 4 Nephrology, Dialysis and Renal Transplant Unit, Department of Pharmacy, Health and Nutritional Sciences, University of Calabria, Rende—Hospital ‘SS. Annunziata’, 87100 Cosenza, Italy

**Keywords:** kidney transplant, rejection, risk factors, graft survival, extended criteria donors

## Abstract

**Background**: Acute rejection (AR) in kidney transplant (KT) recipients remains a significant challenge for short- and long-term graft survival even in the most recent years characterized by extended criteria donors and older and more comorbid recipients. **Methods**: We analyzed risk factors and outcomes of AR in 339 KT recipients treated at St. Orsola-Malpighi Hospital, Bologna (Italy), between 1 January 2019 and 31 December 2021. Demographic, immunological, and transplant data (type, cold ischemia time, complications) were recorded with a follow-up period of up to 24 months. Key outcomes included estimated glomerular filtration rate (eGFR), 24 h proteinuria, delayed graft function (DGF), biopsy-proven AR, and graft loss. **Results**: During the first year after transplant, 57 AR episodes occurred: 19 antibody-mediated rejections (AMR), 18 borderline T cell-mediated rejections (TCMR), 18 TCMR, 2 mixed AMR/TCMR, and 11 graft losses. AR was linked to older donor age (59.9 ± 12.8 vs. 55.5 ± 15.1, *p* = 0.040), longer cold ischemia time (690 vs. 570 min, *p* = 0.044), higher DGF rates (61.40% vs. 39.57%, *p* = 0.002), and lower eGFR (39 vs. 52 mL/min, *p* = 0.003). AR was consistently prevalent in patients who underwent an AB0-incompatible (AB0-i) transplant (8.8% vs. 2.5%, *p* = 0.020). HLA matching was strongly associated with a reduced risk of AMR (HLA-DR: OR 0.35, HLA-A: OR 0.33, HLA-C: OR 0.35), while DGF was linked to a higher risk (OR 4.04). TCMR risk was associated with donor age (OR 1.05). The development of post-transplant donor-specific antibodies (DSAs) at 24 months showed no significant association with AR (AMR: *p* = 0.769; TCMR: *p* = 0.938). The decline in eGFR over time (24 months) did not differ between patients with and without AR (difference, −0.69 mL/min/year; Standard Error, 0.92; *p* = 0.452). Similarly, 24 h proteinuria change over time did not differ between patients with and without AR (difference, −0.12 g/24 h; Standard Error, 0.28; *p* = 0.657). **Conclusions**: Understanding the risk factors of AR is crucial to identifying KTs at more risk of rejection and to guiding targeted therapeutic decisions. In the most recent era of extended criteria donors and more vulnerable recipients, early diagnosis and prompt and tailored treatment of AR play a critical role in stabilizing renal function over time.

## 1. Introduction

Kidney transplants (KTs) are currently the most effective therapeutic option for End-Stage Kidney Disease (ESKD) [1]. KT recipients have a lower mortality rate, a lower risk of cardiovascular events, and an improved quality of life compared to patients remaining on dialysis and with similar comorbidities [2]. The chronic shortage of organs has been faced over the past two decades by extending the pool of donors, like expanded criteria donors (ECD), and through donation after circulatory death (DCD) [3]. Studies have demonstrated significant benefits to receiving a kidney from a marginal donor compared to remaining on dialysis [4,5]; however, this approach is still associated with a higher risk of delayed graft function (DGF) and an increased likelihood of acute rejection (AR) [6]. Additionally, the profile of transplant candidates has evolved, including older recipients and an increased number of re-transplants [7]. Despite these insights, advancements in medical management have resulted in improved short- and long-term grafts and patient survival rates [3]. Further evidence is needed to assess the incidence and outcomes of kidney transplant complications within this new setting. AR continues to be one of the most significant challenges in kidney transplantation [8], and it is linked to an elevated risk of graft loss in both the short- and long-term [9]. Moreover, AR is a risk factor for the development of chronic rejection, particularly when it occurs over 6 months after transplantation [10]. Several clinical predictors of AR have been identified, including recipient age, race, and history of prior transplantation, as well as immunologic factors such as HLA (human leukocyte antigen) matching and pre-transplant Panel Reactive Antibody (PRA) levels [3]. Unfortunately, the current pre-transplant risk stratification does not sufficiently prevent the onset of early AR [11]; therefore, gaining a deeper understanding of these risk factors could aid clinicians in making more informed therapeutic decisions, ultimately improving long-term graft outcomes. When it occurs, only prompt treatment of AR may prevent patients from graft loss. To date, management of AR includes effective treatments, such as high-dose glucocorticoids, anti-thymocyte globulins (ATG), plasma exchange, intravenous immunoglobulins (IVIG), monoclonal antibodies (e.g., rituximab, eculizumab, tocilizumab, daratumumab), and proteasome inhibitors (e.g., bortezomib). The choice of an appropriate therapy is guided by the type of rejection at kidney biopsy (T cell-mediated rejection, TCMR; antibody-mediated rejection, AMR; or mixed rejection) and by the severity of signs and symptoms. Indeed, the selection of an appropriate immunosuppressive therapy should be guided not only by the histological diagnosis of rejection and graft function but also by the risk of over-immunosuppression and infections [12]. Based on this background, we designed a cohort study with the aim of investigating the risk factors of AR in kidney transplant recipients and assessing their prognostic role over time.

## 2. Materials and Methods

This is an observational retrospective cohort study conducted in consecutive kidney transplant recipients enrolled at the St. Orsola Hospital of Bologna between 1 January 2019 and 31 December 2021. Patients receiving repeated transplants during the follow-up were excluded. This study was approved by the Ethics Committee (586/2023/Oss/AOUBo). According to the Italian National Kidney Allocation System, all transplants were performed with negative complement-dependent cytotoxicity (CDC) and/or flow cytometric (FC) crossmatches. Deceased donor transplants - including those from donation after brain death (DBD) and DCD - were all AB0 compatible. Living donor (LD) transplants were both AB0-i- and AB0-compatible, and a low isoagglutinin antibody titer (≤1:8) was considered acceptable for transplantation. As per the Italian National Waiting List protocols, anti-HLA antibodies were repeatedly tested during the stay on the waiting list.

Donor-specific antibody (DSA) monitoring was conducted per protocol monthly for the first six months in immunized recipients, every three months during the first year in non-immunized patients, and then yearly thereafter. DSAs were measured per clinical indication during the entire follow-up post-transplant.

At baseline (discharge from the hospital) and at each follow-up (3, 12, 18, 24 months), we collected patients’ demographical and immunological data, including gender, age at transplant, underlying nephropathy, PRA%, and re-transplantation, along with the donor’s age and the type of donor. The characteristics of donors, recipients, and graft outcomes were considered, including the Karpinski score, cold ischemia time, AB0 compatibility, HLA matching, crossmatch, DGF, primary nonfunction (PNF), serum creatinine, eGFR, 24 h proteinuria, and DSA development. Recipients underwent KT with appropriate immunosuppressive treatment. Induction therapy included basiliximab (20 mg IV on post-operative days (POD) 0 and 4) or ATG (1–1.5 mg/kg on POD 0, 3, and 5 according to absolute lymphocyte count). Maintenance therapy generally included triple immunosuppressive treatment with calcineurin inhibitors (CNIs) or mTOR inhibitors, mycophenolate mofetil (MMF), and corticosteroids. Desensitization therapies for AB0-i living donor transplants were based on anti-CD20 antibody (rituximab), a variable number of immunoadsorption (IA) and/or plasma exchange (PEX) sessions, IVIG, pre-operative oral tacrolimus (FK), and MMF, following our published protocol [13]. We analyzed AR events during the first year after transplant.

In case of rejection, histopathological features at kidney biopsy were reported according to the Banff 2019 classification [14]. The treatments of rejection comprehensively included the following: high-dose glucocorticoids (500 mg/day for three consecutive days) and/or ATG for TCMR, and, depending on the severity and histological characteristics of AMR, plasma exchange, IVIG, and/or rituximab, tocilizumab, or eculizumab.

### Statistical Analysis

Continuous variables were reported as either mean ± standard deviation (SD) or median and interquartile (IQR) range, depending on their distribution. Comparison among rejection risk categories was assessed using the unpaired Student *t*-test or the Kruskal–Wallis test based on variable distribution. Categorical variables were reported as percentages, and comparisons between categories were assessed using the chi-square test. For survival analysis, follow-up was started from the baseline visit (discharge), and its median value was estimated by the inverse Kaplan–Meier approach. Patients lost to follow-up were censored at the time of the last outpatient visit. Next, we computed the adjusted risk for T cell–mediated rejection or antibody-mediated rejection. For the model-building process, univariate analysis testing the association between the main clinical variables and each type of rejection was assessed by means of logistic regression analysis. The variables with *p* < 0.15 for the univariate analysis were selected and included in the first multivariate regression model. Next, a backward variable selection method with an elimination criterion of *p* < 0.10 was performed to fit the final multivariate logistic regression model. A stringent cut-off for variable inclusion was used to avoid model overfitting due to the limited sample of the cohort. Multicollinearity was assessed with variance inflation factors (VIFs), which are a measure of the degree to which a single predictor variable can be expressed as a linear combination of the remaining predictor variables; values greater than 10 were cause for concern. The effects of acute rejection on continuous variables, such as eGFR and proteinuria, were analyzed using a mixed model with repeated measurements with an unstructured covariance matrix to estimate within-subject correlations. A two-tailed *p*-value < 0.05 was considered significant. Data were analyzed using STATA version 14.0 (StataCorp., College Station, TX, USA).

## 3. Results

### 3.1. Baseline Characteristics

We analyzed data from 339 kidney transplant recipients performed at St. Orsola Hospital of Bologna (Italy) between 1 January 2019 and 31 December 2021. The whole population was characterized by a mean recipient age of 51.1 (SD ±13.6) years and a higher frequency of males (62.2 vs. 37.8%). The most prevalent renal diagnoses were glomerulonephritis (31.3%), tubulo-interstitial nephritis (16.5%), and polycystic kidney disease (18.6%). The mean donor age was 56.3 ± 14.9 years, and the mean cold ischemia time was 587 min (80–305). Immunosuppressive induction therapy consisted of basiliximab in 63.5% of patients and ATG in 36.5%. The prevalence of DGF was 43.3% overall (Table 1).

### 3.2. Acute Rejection Characteristics

AR occurred in 57 patients (16.8%); among rejections, 33.3% were AMR, 63.2% TCMR (including 31.6% of borderline TCMR), and 3.5% mixed rejections during the first year after transplant. TCMR occurred at a median follow-up of 0.48 [IQR 0.31–3.04] months from the kidney transplant, whereas AMR developed at 0.44 [IQR 0.33–1.37] months, in median.

Compared with patients without rejection, those with rejection had a significantly longer cold ischemia time (690 vs. 570 min, *p* = 0.044) and a higher prevalence of DGF (61.4% vs. 39.6%, *p* = 0.002). The mean donor age was higher in patients with rejection (59.9 vs. 55.5 years, *p* = 0.040) when compared with those without rejection, and the donor–recipient age difference was also greater in patients with AR (−8 vs. −4 years, *p* = 0.002). AR was consistently prevalent in patients who underwent AB0-i transplants (8.8% vs. 2.5%, *p* = 0.020).

Table 2 shows adjusted analysis for logistic regression of clinical factors associated with AR: increasing number of HLA matching (HLA-DR, HLA-A, and HLA-C) was strongly associated with a reduced risk of AMR, with Odds Ratios (ORs) of 0.35 (95% CI: 0.15–0.85), 0.33 (95% CI: 0.12–0.87), and 0.35 (95% CI: 0.13–0.96), respectively, while DGF was linked to a higher risk (OR 4.04, 95% CI: 1.32–12.31). For TCMR, donor age significantly increased AR risk (OR 1.05, 95% CI: 1.01–1.08). The chi-square test revealed no statistically significant association between AMR (χ2 = 0.0863, *p* = 0.769) or TCMR (χ2 = 0.006, *p* = 0.938) and the development of post-transplant DSAs at the 24-month follow-up (Table 3). Moreover, neither AMR nor TCMR demonstrated any statistically significant association with the development of post-transplant DSAs in the Cox regression analysis (*p* > 0.05) (Table 4).

### 3.3. Kidney Measures (eGFR and Proteinuria)

**Figure 1:**
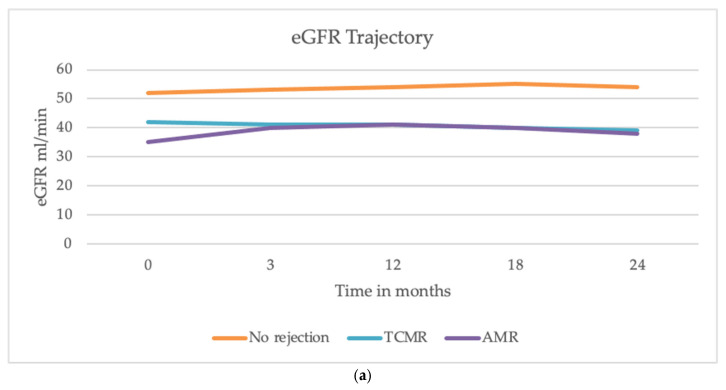

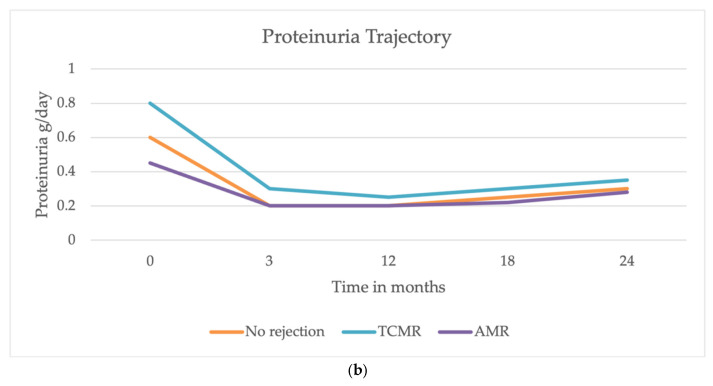
Trajectories of eGFR and proteinuria over 24 months after the transplant. (**a**) The decline in eGFR was similar among patients with AMR, TCMR, and those without AR. (**b**) The trajectory of proteinuria showed a decline in all groups during the first year, with a slight increase observed between 18 and 24 months. No significant differences were observed in proteinuria trends between patients with and without AR.

Although at baseline a statistically significant difference was observed (eGFR 39 vs. 52 mL/min, *p* = 0.003), during the follow-up GFR decline was not different between patients with and without AR (difference, −0.69 mL/min/year; Standard Error, 0.92; *p* = 0.452) (Figure 1a). Similarly, 24 h proteinuria change over time did not differ between patients with and without AR (difference, −0.12 g/24 h; Standard Error, 0.28; *p* = 0.657, Figure 1b).

## 4. Discussion

Our study provides insights into the risk factors and outcomes of AR in a population of kidney transplant recipients. Biopsy-proven AR occurred in 16.8% of patients, and it was significantly associated with higher donor age, increased cold ischemia time, increased frequency of DGF, AB0 incompatibility, and donor–recipient age difference. These findings are consistent with the existing literature [9,15], but their prognostic role among the modern population of kidney transplant recipients is still a matter for research. Wu et al. demonstrated the association among DGF and biopsy-proven AR in a modern cohort of 645 KT recipients over 12 years [6]. Indeed, DGF remains one of the major and frequent complications affecting >25% of KT recipients from deceased donors [3], leading to early acute kidney injury, worsening renal outcomes, and increasing rejection rates [16]. The adjusted logistic regression analysis reveals a significant association between DGF and the occurrence of acute AMR in our population, and acute tubular necrosis appears to be potentially associated as well. Therefore, despite recent advancements in the use of hypothermic perfusion machines, ischemia-reperfusion injury is still unavoidable, and new pre-transplant tools to predict the development of DGF after surgery are urgently needed.

Donor age was also significantly associated with an increased risk of acute TCMR; however, donor age and donor–recipient age difference are still a matter of discussion among experts in the transplant field. The use of older living kidney donors appears to be beneficial compared to remaining on a waiting list for older deceased donors [17], but the impact of age difference on transplant outcomes remains controversial [18]. In our population, the donor–recipient age difference was higher among patients with AR; the awareness of such a risk factor can guide the clinician in selecting an immunosuppressive regimen tailored to the patient’s specific risk profile.

All our patients were transplanted in the presence of a negative crossmatch, and among HLA antigen matches, the increasing number of HLA-DR, -A, and -C matches is associated with a lower risk of rejection at the multivariate analyses. It is well known that pre-transplant matching of HLA-DR antigens has a beneficial role in improving graft survival [19,20], and previous studies have stressed the critical role of class I HLA antigen matching in first transplants for patients who may require future re-transplantation [21]. In the past decade, developments in the pre-transplant immunologic assessment have been reached and new and more sophisticated signatures to predict kidney transplant rejection are currently under validation. In the future, the aim is to develop new algorithms based on several immunologic factors and genetic/epigenetic signatures that may provide risk stratification with a significant predictive value.

Furthermore, we evaluated if the incidence of rejection could be significantly associated with an increased risk of DSA development post-transplant. Our results show no significant correlation between kidney rejection and the subsequent development of DSAs at the 24-month follow-up. Previous studies suggest that AR promotes the production of antibodies against donor antigens, thus increasing the risk of new episodes. The mechanisms underlying this correlation appear multifaceted and are not completely defined. It is known that during AR, there is an upregulation of pro-inflammatory cytokines and an increased presentation of donor antigens by antigen-presenting cells. This inflammatory environment enhances T and B cell activation, thereby promoting the generation of DSAs. Additionally, endothelial injury and subsequent repair processes during rejection may expose cryptic antigens, further stimulating the immune response [22]. Nevertheless, our population did not show a direct association between rejection and post-transplant DSA development, although a 2-year follow up is not sufficient to define this risk in the long term, and we considered positive DSAs with MFI > 3000. Further studies with a longer follow-up are needed to determine whether the rejection event is significantly associated with an increased risk of developing chronic or chronic-active rejection (with or without positive DSAs). Moreover, anti-rejection therapy is essential for stabilizing graft function over time, as we demonstrated in our cohort: we found that the decline in eGFR did not differ significantly between patients with and without AR, including both AMR and TCMR (Figure 1a). Similarly, proteinuria trajectory did not significantly differ between those who developed rejection and those who did not over two years (Figure 1b) at the 24-month follow-up. These findings align with recent studies reporting that AR episodes may not have an immediate and pronounced impact on eGFR and proteinuria [23], and that a prompt treatment of AR may significantly impact graft outcomes in terms of kidney function. Indeed, recent evidence suggests that while AR episodes are critical, their immediate effects on graft function might be mitigated by timely and effective anti-rejection therapies. Studies have demonstrated that high-efficacy immunosuppressive regimens can effectively control rejection and stabilize graft function, emphasizing the importance of early intervention [24,25]. Furthermore, the increasing recognition of biomarkers in predicting and monitoring rejection offers potential for more tailored therapeutic approaches, even though prevention remains the main goal to mitigate kidney damage. Despite these insights, biomarkers for early diagnosis of AR and optimal treatment strategies to improve graft function and long-term outcomes remain areas of research. Our study has some limitations: a small sample size and the inclusion of patients in only one center limit the generalizability of results to other centers and countries. Future studies can confirm the results. Moreover, the small number of rejection events, despite being relevant in a cohort of kidney transplant recipients, makes it not possible to run more sophisticated analyses, such as time-to-rejection event.

## 5. Conclusions

This study investigates the predictors of acute rejection in a cohort of kidney transplant recipients, focusing on their impact on graft functional outcomes. Our findings highlight the significant roles of clinical factors such as DGF, older donor age, prolonged cold ischemia time, and HLA matches in predicting acute rejection. Identifying these risk factors provides critical insights for therapeutic decision-making, especially given the current lack of reliable biomarkers.

Encouragingly, the stable eGFR trajectory in patients with acute rejection highlights the potential effectiveness of modern immunosuppressive therapies. This stabilization is particularly notable within the current clinical setting, characterized by the increased use of marginal donors and recipients with heightened vulnerability. These results highlight the progress being made in managing acute rejection, even in challenging scenarios. Future research should prioritize the refinement of risk stratification strategies, the identification of early biomarkers of rejection, and the development of tailored immunosuppressive regimens. Ultimately, these efforts aim to enhance vigilant monitoring and enable timely interventions to preserve graft function and improve long-term patient outcomes.

## Figures and Tables

**Table 1:** Basal characteristics of patients: overall and by RI risk categories.

	Overall (*n* = 339)	Rejection: Yes (*n* = 57)	Rejection: No (*n* = 282)	*p*-Value
Age (recipient), years	51.1 ± 13.6	49.9 ± 12.5	51.3 ± 13.8	0.475
Age (donor), years	56.3 ± 14.9	59.9 ± 12.8	55.5 ± 15.1	0.040
Age difference, years	−4 [−13–0]	−8 [−15–−3]	−4 [−11–1]	0.002
Male gender, %	62.2	61.4	62.4	0.886
Renal diagnosis				0.089
Hypertensive Nephropathy	6.2	3.5	6.7	
Diabetes	3.8	7.0	3.2	
Glomerulonephritis	31.3	21.1	33.3	
Tubulo-interstitial Nephritis	16.5	19.3	16.0	
Polycystic Kidney Disease	18.6	28.1	16.7	
Unknown	20.1	21.1	19.9	
Others	3.5	0	4.3	
PRA max, %	5 [0–56]	5 [0–61]	3 [0–55]	0.513
AB0-i, %	3.6	8.8	2.5	0.020
Basiliximab vs. Thymoglobulins				0.399
Thymoglobulins, %	36.5	31.5	37.6	
Basiliximab, %	63.5	68.5	62.5	
Karpinski score, mean	3.5 ± 1.4	3.4 ± 1.3	3.6 ± 1.4	0.558
Cross-match B	15.9	22.8	14.5	0.118
N° HLA-A Matches, % ^1^				0.498
0	37.3	43.9	35.9	
1	51.5	47.4	52.3	
2	11.2	8.8	11.7	
N° HLA-B Matches, %				0.232
0	53.9	47.4	55.2	
1	37.9	47.4	35.9	
2	8.3	5.3	8.9	
N° HLA-C Matches, %				0.621
0	42.0	38.2	42.8	
1	48.8	54.6	47.6	
2	9.2	7.3	9.6	
N° HLA-DQ Matches, %				0.345
0	12.3	15.8	11.6	
1	57.5	61.4	56.7	
2	30.2	22.8	31.8	
N° HLA-DR Matches, %				0.450
0	24.3	28.1	23.5	
1	58.0	59.7	57.7	
2	17.8	12.3	18.9	

^1^ Odds ratios are reported per additional matched allele.

**Table 2:** Logistic regression analysis of clinical factors associated with AR, including AMR and TCMR. Results are reported with a 95% confidence interval (CI).

AMR	Odds Ratio (95% CI)	*p*-Value
Match HLA-DR	0.35 (0.15–0.85)	0.021
Match HLA-A	0.33 (0.12–0.87)	0.025
Match HLA-C	0.35 (0.13–0.96)	0.041
DGF	4.04 (1.32–12.31)	0.014
**TCMR**		
Age (recipient), years	0.97 (0.94–1.00)	0.068
Age (donor), years	1.05 (1.01–1.08)	0.010
ATN	2.57 (0.99–6.69)	0.054

**Table 3:** Chi-square test to assess the association between AR episodes (AMR and TCMR) and the development of DSAs at the 24-month post-transplant follow-up.

AMR	No DSA	DSA ^1^	Total
No	281	37	318
Yes	19	2	21
Total	300	39	339
**TCMR**			
No	268	35	303
Yes	32	4	36
Total	300	39	339

^1^ DSAs were considered positive only if the Mean Fluorescent Intensity (MFI) exceeded 3000.

**Table 4:** Cox regression analysis results evaluating the association between AMR, TCMR, and the development of post-transplant DSAs at the 24-month follow-up post-transplant.

Rejection Type	Hazard Ratio (HR) (95% CI)	*p*-Value
**AMR**	0.86 (0.21–3.59)	0.835
**TCMR**	0.99 (0.35–2.81)	0.989

## Data Availability

The data presented in this study are available on request from the corresponding author due to privacy/ethical restrictions.

## Abbreviations

The following abbreviations are used in this manuscript:

AR	acute rejection
KT	kidney transplant
TCMR	T cell-mediated rejection
AMR	antibody-mediated rejection
eGFR	estimated glomerular filtration rate
ECD	expanded criteria donors
DCD	donation after circulatory death
DGF	delayed graft function
HLA	human leukocyte antigens
PRA	panel reactive antibodies
DSA	donor specific antibody
CDC	complement-dependent cytotoxicity
FC	flow cytometric
POD	post-operative day
ATG	anti-thymocyte globulins
IVIG	intravenous immunoglobulins
MMF	mycophenolate mofetil
